# Enhancing the graphene photocurrent using surface plasmons and a p-n junction

**DOI:** 10.1038/s41377-020-00344-1

**Published:** 2020-07-20

**Authors:** Di Wang, Andres E. Llacsahuanga Allcca, Ting-Fung Chung, Alexander V. Kildishev, Yong P. Chen, Alexandra Boltasseva, Vladimir M. Shalaev

**Affiliations:** 1 School of Electrical and Computer Engineering, Purdue University, West Lafayette IN, 47907 USA; 2 Birck Nanotechnology Center, Purdue University, West Lafayette IN, 47907 USA; 3 Department of Physics and Astronomy, Purdue University, West Lafayette IN, 47907 USA; 4 Purdue Quantum Science and Engineering Institute (PQSEI), Purdue University, West Lafayette IN, 47907 USA

**Keywords:** Imaging and sensing, Photonic devices, Metamaterials, Optoelectronic devices and components, Optical sensors

## Abstract

The recently proposed concept of graphene photodetectors offers remarkable properties such as unprecedented compactness, ultrabroadband detection, and an ultrafast response speed. However, owing to the low optical absorption of pristine monolayer graphene, the intrinsically low responsivity of graphene photodetectors significantly hinders the development of practical devices. To address this issue, numerous efforts have thus far been made to enhance the light–graphene interaction using plasmonic structures. These approaches, however, can be significantly advanced by leveraging the other critical aspect of graphene photoresponsivity enhancement—electrical junction control. It has been reported that the dominant photocarrier generation mechanism in graphene is the photothermoelectric (PTE) effect. Thus, the two energy conversion mechanisms involved in the graphene photodetection process are light-to-heat and heat-to-electricity conversions. In this work, we propose a meticulously designed device architecture to simultaneously enhance the two conversion efficiencies. Specifically, a gap plasmon structure is used to absorb a major portion of the incident light to induce localized heating, and a pair of split gates is used to produce a p-n junction in graphene to augment the PTE current generation. The gap plasmon structure and the split gates are designed to share common key components so that the proposed device architecture concurrently realizes both optical and electrical enhancements. We experimentally demonstrate the dominance of the PTE effect in graphene photocurrent generation and observe a 25-fold increase in the generated photocurrent compared to the un-enhanced cases. While further photocurrent enhancement can be achieved by applying a DC bias, the proposed device concept shows vast potential for practical applications.

## Optics: designing lightweight, broadband graphene photodetectors

American scientists have developed graphene photodetectors with unprecedented compactness, ultra-broadband detection, and ultrafast response speeds. In addition to being inexpensive, lightweight, and compact, graphene has unique optical and electrical properties, including a zero bandgap, ultrahigh carrier mobility, and thermal conductance that make it ideal for use in photodetectors. However, as graphene is just one monolayer, it absorbs light very weakly, thus hindering the development of practical photodetectors. A team of researchers led by Vladimir Shalaev from Purdue University in the United States has developed a plasmonic-enhanced graphene photodetector with a 25-fold increase in photocurrent generation compared with conventional graphene devices. Moreover, further enhancement can be achieved by the application of a direct current bias, opening the door for useful applications that require small devices with broad operational bandwidths and excellent responsivity.

## Introduction

Since its first successful isolation from bulk graphite, graphene has been extensively studied as a photodetection material^1^. In addition to being cheap, lightweight and compact, graphene has a number of optical and electrical signatures that make it a unique photodetection material. Specifically, graphene offers (i) unlimited detectable wavelength range owing to the zero bandgap^2^, (ii) uniform responsivity over the entire spectrum, resulting from the invariant optical absorption (2.3%)^3^, and (iii) an ultrafast response speed because of the ultrahigh carrier mobility (for the photovoltaic (PV) effect)^4,5^ and thermal conductance (for the photothermoelectric (PTE) effect)^6–9^. Despite these remarkable features, the relatively low responsivity (defined as the photocurrent amplitude per input optical power, in A/W) significantly hinders practical applications of graphene photodetectors. This low responsivity is mainly owing to the weak absorption of light by single layer graphene^10^. Therefore, various systems have been investigated to assist light-graphene interactions and enhance the responsivity. Among them, the quantum-dot-loaded graphene phototransistor exhibits the highest responsivity gain^11,12^, but the strong gain comes at the cost of a reduced operation speed (~10 ms, or 100 Hz) owing to the slow quantum dot discharge process. Photonic waveguides have also been utilized to boost the responsivity; however, such structures usually have large and sophisticated device footprints because long waveguides are required to increase the photon-graphene interaction length^13,14^. A third approach to enhance the graphene photoresponsivity is to utilize plasmonic structures, which relies on the highly confined light-induced surface plasmon oscillations in metallic nanostructures to aid light absorption in graphene^15–19^. Such systems have a relatively simple device architecture and do not compromise the graphene photodetector operation speed. However, most of the proposed plasmonic-enhanced graphene photodetectors utilize only optical enhancement, whereas electrical junction control (discussed in detail later) is largely neglected. In this article, we propose a system that utilizes both optical and electrical control of a plasmonic-enhanced graphene photodetector and shows superior performance compared to previously suggested designs.

## Results

**Fig. 1:**
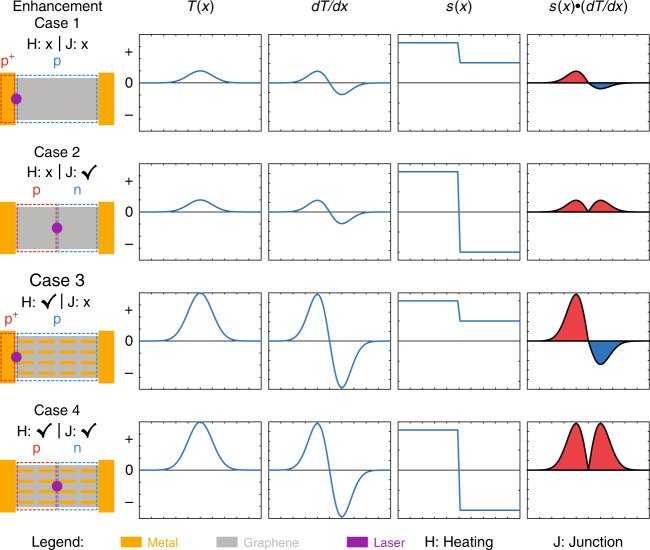
Schematic illustration of the photothermoelectric (PTE) current generation in graphene. Based on the enhancement conditions of optical heating and an electrical junction, graphene photodetectors can be categorized into four cases, each represented by a row. In all plots, the horizontal axes denote the spatial coordinate *x*, and the vertical axes are denoted by their respective column headers. The T(*x*) column illustrates the electron temperature profile owing to laser illumination, and the dT/d*x* column is obtained by taking the spatial derivative of T(*x*). *s*(*x*) represents the spatial distribution of the Seebeck coefficient, and the *s*(*x*)∙(dT/d*x*) column is obtained by taking the product of the *s*(*x*) and dT/d*x* columns. The PTE current is calculated by integrating the *s*(*x*)∙(dT/d*x*) function, i.e., adding the areas of the shaded regions, with red (blue) denoting the positive (negative) contribution to the PTE current

The key factor in improving the responsivity of graphene photodetectors is enhancing the PTE effect that separates the free charge carriers via a temperature gradient ($\nabla$T). This concept originates from earlier works, demonstrating that the PTE effect is the dominant photocarrier generation mechanism in graphene^20–23^. In simple words, the majority of charge carriers (electrons or holes, depending on the doping type of graphene) are driven from the hot region to the cold region, and the net charge carrier movement leads to a detectable photocurrent. Typical graphene photodetectors rely on optically induced local heating to generate a PTE current. However, intrinsic graphene sheets convert only 2.3% of incident light to heat, leaving much room for improvement. Plasmonic systems—devices that harness the optically induced unbound electron oscillations in metallic scatterers to enable nanoscale light control^24–27^—are promising candidates to generate localized heating, thereby a large T, upon optical illumination^28–30^. However, equally important in graphene photocurrent generation is the uneven electrical doping level (i.e., a junction) at the centre of the $\nabla$T to give rise to a nonzero photocurrent^31^. Otherwise, if the doping level is uniform, then the same type of charge carrier would be driven by the $\nabla$T in opposite directions with the same strength, resulting in zero net current. In most plasmonic-enhanced graphene photodetectors, doping is achieved passively by depositing metal contacts on graphene^32^, and uneven doping, thereby the maximum photocurrent, occurs at the metal contact edges (see Fig. 1 first row). The majority of previously reported approaches do not offer control over the graphene doping level because the doping level in the metal-contacted graphene region is fixed. In this work, we introduce doping control in addition to the widely used optical enhancement via plasmonic structures. Because doping is achieved via electrical gating, we henceforth refer to doping control/enhancement as electrical or junction control/enhancement.

As reported in ref. ^20^, the PTE effect largely dominates the photocurrent generation mechanism in graphene with channel lengths >5 μm. Thus, in this work, we neglect the competing PV effect (the channel length in our device is 50 μm). The PTE current in graphene is governed by

1 $$I_{PTE} ={\int\nolimits_0^W} {{\int \nolimits_{ - L/2}^{L/2}} {s\left( x \right)\nabla T_{el}\frac{{{\mathrm{d}}x{\mathrm{d}}y}}{{RW}}} }$$

where *W* and *L* are the width and length of the graphene sheet, respectively, *s*(*x*) is the spatial distribution of the Seebeck coefficient (controlled by the doping type and level in graphene), *T*_*el*_ is the electron temperature, and *R* is the total resistance of graphene. To better illustrate the graphene PTE current generation mechanism, we simplify Eq. 1 by considering only one dimension (along the *x* direction) and create a series of schematic illustrations in Fig. 1. The illustrations are categorized into four cases (rows), depending on the optical heating and electrical junction enhancement scenarios. The last column of Fig. 1 depicts the integrand of Eq. 1, and the PTE current is directly obtained by calculating the area under each curve, with the red-shaded area denoting the positive contribution and the blue-shaded area denoting the negative contribution. Case 1 represents the most pristine graphene photodetectors, which consist of only metal contacts and graphene, with neither heating nor junction enhancement^33,34^. Note that both the metal contacts and common fabrication-induced contaminants p-dope graphene; thus, *s*(*x*) is positive in both the metal-contacted region and exposed region and exhibits a step owing to the difference in the doping levels. As a result, the integrand has unequal positive and negative contributions, resulting in a nonzero PTE current. Some previous works have studied the graphene PTE effect using vigorous electrical junction control without plasmonic enhancement^21,22,35^, thus belonging to Case 2. Here, a p-n junction is introduced at the centre of the temperature gradient, which ensures a positive contribution to the PTE current on both sides. As mentioned above, plasmonic structures help convert light into local heating (represented by high T(*x*) and dT/d*x* profiles in Fig. 1); hence, most of the previously demonstrated plasmonic-enhanced graphene photodetectors^16–18^ fall into Case 3. It is evident that Case 4 with both optical heating and electrical junction enhancements results in the strongest PTE current, which forms the backbone of the design presented in this work.

**Fig. 2:**
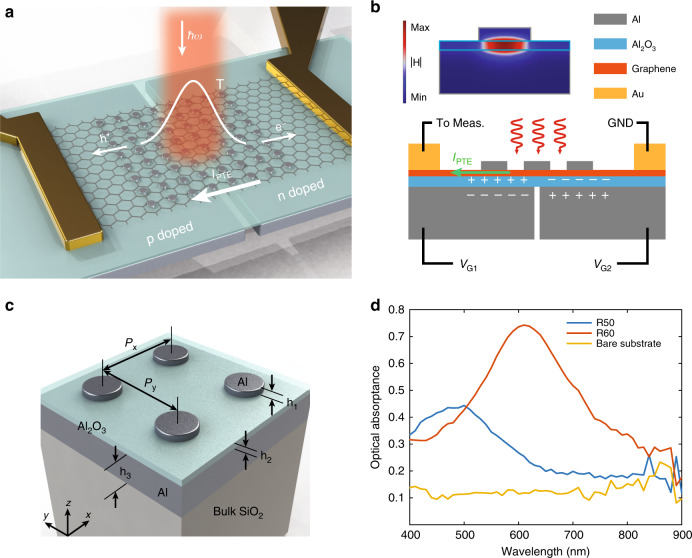
Graphene photodetector design and optical characterization. **a** Schematic of the graphene photodetector that integrates both optical heating enhancement (realized via a gap plasmon structure) and electrical junction enhancement (realized via split gates, channel length = 50 μm, width = 30 μm, central gap = 150 nm). The graphene lattice is not drawn to scale. **b** Cross-sectional schematic of the graphene photodetector with split gates and a nanodisk gap plasmon structure. *V*_G1_ and *V*_G2_ are used to independently control the doping types on the two sides of graphene. The nanodisks cause localized heating by absorbing light, which drives electrons (holes) from the centre to the left (right) to give rise to *I*_PTE_. The top left inset shows the simulated normalized magnetic field distribution under resonant conditions, where most of the incoming electromagnetic energy dwells inside the Al_2_O_3_ layer instead of being reflected back. The nanodisk, Al_2_O_3_, and Al reflector layers are outlined by their representative colours. GND: ground. **c** Schematic of the gap plasmon structure: aluminium (Al) nanodisk array (NDA). The Al nanodisks have a thickness of *h*_1_ = 30 nm, are arranged with periodicity *P*_x_ = *P*_y_ = 300 nm, and reside on an Al_2_O_3_ layer with a thickness of *h*_2_ = 20 nm atop an Al back reflector layer with a thickness of *h*_3_ = 100 nm. The disk radius varies across the NDAs, and all the arrays are deposited on a 0.7 mm thick bulk SiO_2_ substrate. **d** Measured optical absorptance spectrum in the range of 400–900 nm on two NDAs with *R* = 50 nm and *R* = 60 nm and on a bare substrate (only Al_2_O_3_ and Al layers on bulk SiO_2_) without nanodisks. The absorptance measurements are carried out on samples containing no graphene

Figure 1 illustrates the importance of the spatial overlap between the centre of the optical heating area and a p-n junction for maximal graphene photocurrent generation. In this work, we create the p-n junction in graphene using a pair of split gates formed by (from bottom to top) an aluminium (Al)-aluminium oxide (Al_2_O_3_)-graphene parallel-plate capacitor structure and deposit Al nanodisks on graphene to form a gap plasmon structure with the underlying Al_2_O_3_ and Al layers to enhance the optical absorptance. In this way, the optical heating enhancement (gap plasmon structure) and the electrical enhancement (split gates) are seamlessly combined through their shared components of the Al_2_O_3_ and Al layers. More importantly, gap plasmon structures have been shown to exhibit high optical absorptance^36–38^ and are especially efficient in light-to-heat conversion^39^. The choice of materials is based on two reasons: (i) it is relatively easy to tune the resonance wavelength of the Al gap plasmon structure in the visible spectrum to make colour-sensitive graphene photodetectors; (ii) the high quality of Al_2_O_3_ grown on Al guarantees robust electrical gating of graphene. In this work, we investigate the realistic optimal responsivities obtainable in graphene photodetectors that can be produced on a large scale, thus choosing to work with graphene grown by chemical vapor deposition (CVD) because of its compatibility with industry-level fabrication. Figure 2a depicts a schematic illustration of the proposed device and its working principle. A pair of split gates is separated by a tiny gap to ensure electrical isolation between the two gates so that the doping levels on both sides can be controlled independently. As the doping level directly governs the Seebeck coefficient in graphene, we can manipulate the Seebeck coefficients on both sides and create various junction types by electrical gating. Simultaneously, the nanodisk gap plasmon structure absorbs the incident light and creates a localized temperature profile, which then drives the majority charge carriers on the two sides (electrons on the n-doping side and holes on the p-doping side) in opposite directions, leading to a maximized photocurrent. By utilizing a finite element method (FEM) numerical model, we estimated the power dissipation in graphene to be ~14% of the total dissipated power (see Supplementary Information section “Numerical modelling of graphene” for details of the numerical model), which is much higher than that of a graphene sheet without any plasmonic enhancement (~2%). Figure 2b shows a cross-sectional view of the design and illustrates how the split gates and the gap plasmon structure function together. We note that in the fabricated sample, the poor adhesion of nanodisks to graphene somewhat compromises the uniformity of the nanodisk distribution, but this issue can be resolved by introducing a thin Al_2_O_3_ layer between the nanodisks and graphene (not investigated in this work).

As the plasmonic structure has more stringent requirements on the layer thicknesses than electrical gating, the bottom-most Al layer and the Al_2_O_3_ layer are carefully designed to be 100 nm and 20 nm thick, respectively, to achieve high-optical absorptance in the visible spectrum. The split gates are defined by electron-beam lithography (EBL) with a 150 nm wide gap (taking into account the fabrication limitations), Al metallization and lift-off, followed by atomic layer deposition (ALD) growth of Al_2_O_3_. A graphene sheet grown by CVD is transferred onto the Al_2_O_3_ layer, and 30 nm thick Al nanodisks are then formed on the graphene sheet by EBL, metallization and lift-off. See the Materials and Methods section and Supplementary Information Fig. S1 for detailed fabrication procedures. Figure 2c presents a close-up schematic view of the gap plasmon structure without the graphene sheet. The nanodisk radius is varied across samples to alter the absorptance spectrum, thereby controlling the responsivity at different wavelengths. We also fabricate a separate sample containing only the gap plasmon structure (i.e., no graphene) for optical characterization because the absorptance measurement requires an area of uniform gap plasmon structure larger than that of the graphene photodetector. In Fig. 2d, we show three spectra measured on three different areas, one without nanodisks (i.e., only 20 nm thick Al_2_O_3_ and 100 nm thick Al) and two with nanodisks of radii *R* = 50 nm and *R* = 60 nm, and use Fig. 2d as a guideline for the optical absorptance in the nanodisk-loaded graphene photodetectors. Based on the conclusions from ref. ^39^, on the mesoscopic scale, the optically induced $\nabla$T in the gap plasmon structure scales linearly with its optical absorptance. Hence, the responsivity of the gap-plasmon-assisted graphene photodetector is expected to follow the optical absorptance.

## Discussion

**Fig. 3:**
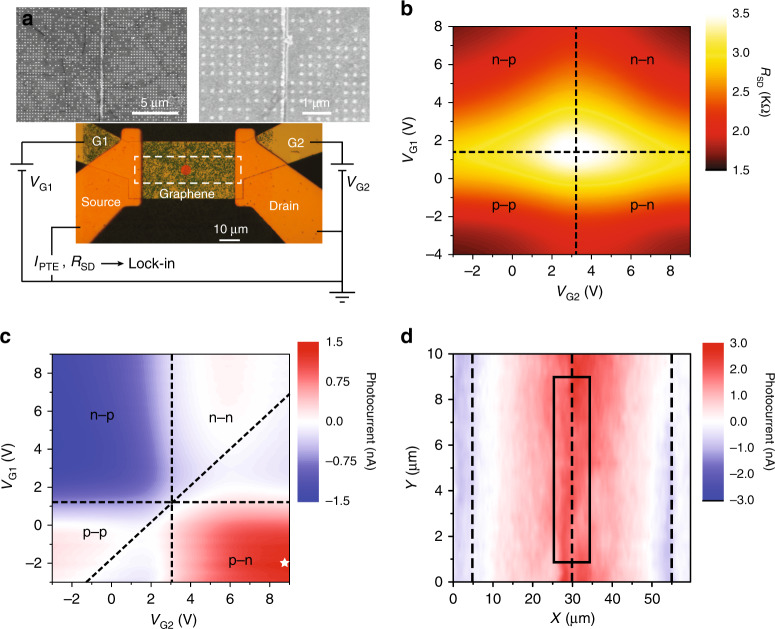
Photocurrent characterization. **a** Microscopic image of a fabricated graphene photodetector loaded with nanodisks with radius *R* = 60 nm, and schematic for the electrical measurements. The green shade results from the strong absorption of the red part of the visible spectrum by the nanodisks. *V*_G1_ and *V*_G2_ are the gating voltages supplied to the two sides of graphene separated by a 150 nm wide gap in the bottom-most Al layer, and the graphene photocurrent signal is measured using a current preamplifier and a lock-in amplifier via the source and drain contacts. The graphene resistance is obtained separately by measuring the voltage drop across the source and drain contacts when a 100 nA current modulated at 17.35 Hz is supplied to the graphene. The two insets show scanning electron micrographs (SEMs) of the fabricated sample. **b** Measured graphene electrical resistance (*R*_SD_) when *V*_G1_ and *V*_G2_ are independently swept. Four junction regimes are identified around the CNP. **c** Measured photocurrent signal as *V*_G1_ and V_G2_ are independently swept, where the dashed lines mark the photocurrent sign changes. The CW laser for this measurement has a power of 32 μW and a diameter of ~10 μm, and its illumination position is marked by the red spot in **a**. **d** Spatially resolved photocurrent map in the area marked by the dashed box in **a**, measured with *V*_G1_ = −2 V and *V*_G2_ = 9 V (indicated by the star in **c**). The vertical dashed lines denote the source/drain contacts and the central 150 nm wide gap, respectively. The data points in the boxed region are used to calculate the average responsivity of this device

We fabricate electrical contacts for electrical characterization under vacuum and room temperature conditions. Figure 3a shows a microscopic image of the fabricated device with *R* = 60 nm nanodisks, as well as the electrical connections made to the device for graphene electrical resistance (*R*_SD_) and photocurrent (*I*_PTE_) measurements. We first measure *R*_SD_ while independently sweeping the voltages *V*_G1_ and *V*_G2_ on the split gates and show the results in Fig. 3b. The central maximum *R*_SD_ point indicates the charge neutrality point (CNP), and two orthogonal lines intersecting at the CNP can be drawn to divide the gating condition into four different regimes based on the types of carrier supplied to either side of graphene. The asymmetry in the two gating voltages at the CNP is likely owing to the trapped charges in the Al_2_O_3_ layer introduced during the fabrication processes. Next, for the *I*_PTE_ measurement, a continuous-wave (CW) laser with *λ* = 638 nm and a power of 32 μW is focused to a spot size of ~10 μm, is modulated by a mechanical chopper at 651 Hz, and illuminates the central area of the photodetector (red spot in Fig. 3a). *I*_TPE_ is then collected from the source and drain contacts using a lock-in amplifier synchronized with the mechanical chopper. Figure 3c shows the measured *I*_PTE_ when *V*_G1_ and *V*_G2_ are swept, from which distinct features can be seen in the four different doping regimes defined in Fig. 3b. In addition, a diagonal line can be drawn along the zero-*I*_PTE_ line in the p-p and n-n doping regimes. The three dashed lines divide Fig. 3c into six regions exhibiting alternating signs of *I*_PTE_, which clearly indicates the dominance of the PTE effect in the photocurrent generation mechanism^20,22^. It is also evident that *I*_PTE_ is strongest under the p-n or n-p doping regime, in agreement with our previous discussion. Finally, we measure the *I*_PTE_ spatial map in the area outlined by the dashed box in Fig. 3a while keeping the gating voltages at *V*_G1_ = −2 V and *V*_G2_ = 9 V (marked by the star in Fig. 3c). As seen from Fig. 3d, *I*_PTE_ is maximum at the central line where the p-n junction is created by the split gates and decreases as the laser moves away from this line. *I*_PTE_ changes sign at the source and drain contact edges owing to the uneven doping between the metal-doped graphene and electrostatically doped graphene (i.e., p(Au)-p^+^(G1) on the source side and n(G2)-p(Au) on the drain side). We calculate the average *I*_PTE_ from the gap plasmon-assisted graphene photodetector by taking the average of the measured data points inside the box highlighted in Fig. 3d. The measured responsivity is 52 μA/W on the device with nanodisks of radius *R* = 60 nm, which has an optical absorptance of 71% at the incident laser wavelength of 638 nm.

**Fig. 4:**
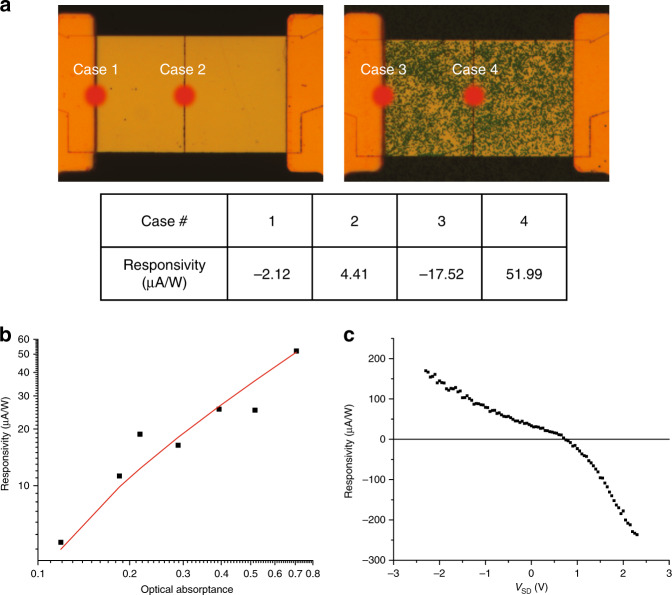
Graphene photodetector enhancement. The four cases categorized in Fig. 1 can be reproduced in the experiment by illuminating the four positions in **a** with the laser spot. In all four measurements, both devices are gated to achieve a p-p^+^ (p-n) junction on the left edge (in the centre) to represent the un-enhanced (enhanced) electrical junction. The device shown on the left (right) contains no nanodisks (nanodisks of *R* = 60 nm) and therefore represents the case of un-enhanced (enhanced) optical heating. The table summarizes the measured photoresponsivities of the four cases, with case numbers corresponding to the enhancement schemes in Fig. 1. **b** Measured photoresponsivity versus optical absorptance (black data points), and linear fit (red line) to the data points. The linear fit appears to be curved owing to the log–log plot scale. The parameters of each measured data point (i.e., disk size, laser wavelength, optical absorptance, and measured responsivity) can be found in the Supplementary Information. All measurements are performed with *V*_G1_ = −2 V, *V*_G2_ = 9 V, and *V*_SD_ = 0. **c** Measured photoresponsivity as a function of the applied DC source-drain bias (*V*_SD_) on a device with an optical absorptance of 71% at an illuminating laser wavelength of 638 nm. All measurements in **b**, **c** are carried out by illuminating the central p-n junction with the laser, and those in **b**, **c** are measured under two different experimental settings

To compare the four cases presented in Fig. 1, we measure the photocurrent in different locations of two separate devices both gated to achieve a p-n junction in the centre and show the results in Fig. 4a. One of these devices contains only a graphene sheet (top left in Fig. 4a), and the other is loaded with nanodisks with radius *R* = 60 nm (top right in Fig. 4a). In both devices, laser illumination of the central line corresponds to the cases of the enhanced p-n junction, whereas illumination of the Au electrode/graphene interface corresponds to an un-enhanced junction, as seen from the reversed sign (because of the p(Au)-p^+^(G1) doping on the left edge and p(G1)-n(G2) doping at the centre) and lower *I*_PTE_ amplitude at the edges of Fig. 3d compared to the central area. On the other hand, the nanodisks dramatically increase the optical absorption, thereby $\nabla$T, making Device 2 represent the cases of enhanced optical heating. The table in Fig. 4a summarizes the measured responsivities for the four different cases. The absolute values of the responsivities correlate well with the schematic illustrations in Fig. 1, strongly substantiating our prediction as well as demonstrating the power of $\nabla$T and junction enhancement. By comparing the photocurrents of Case 4 and Case 1, we conclude that a 25-fold responsivity increase can be achieved with optical and electrical enhancement compared to the generic (un-enhanced) case.

We then repeat the measurement using two additional laser wavelengths (532 and 825 nm) on three devices (no nanodisks and disks with radius *R* = 50 nm and *R* = 60 nm). These measurements allow us to investigate the correlation between the optical absorptance and the *I*_PTE_ responsivity. In Fig. 4b, we plot the average responsivity calculated in the same way as for Fig. 3d versus the measured optical absorptance (see Supplementary Fig. S2 for the measured responsivity maps for all the data points). The red fitting line (note that the red line is a linear fit but appears curved owing to the log–log plot scale and the *y* axis starting from 3 μA/W) indicates that the responsivity scales linearly with the optical absorptance, agreeing with our previous discussion and design principle that the optically induced $\nabla$T, hence responsivity, is directly proportional to the optical absorptance. In addition, the source-drain bias (*V*_SD_) can be utilized to enhance the responsivity by creating a static electric field between the two electrodes to facilitate electron harvesting. Figure 4c presents the measured responsivity as *V*_SD_ is swept from −2.3 to 2.3 V, from which it can be concluded that an additional sevenfold responsivity enhancement can be achieved at 2.3 V compared to zero *V*_SD_. All measurements except the one shown in Fig. 4c are performed with *V*_SD_ = 0 V.

The responsivity can be further enhanced if the following two methods are implemented together with the proposed device architecture: (i) increase the optical absorptance by optimizing the plasmonic system design or implementing photonic crystals^40^ and (ii) improve the quality of graphene. In this work, the highest optical absorptance achieved is 71%. The optical absorptance can be improved by refining the fabrication process, for example, by introducing a thin Al_2_O_3_ layer between the nanodisks and graphene to increase the adhesion between the nanodisks and graphene. As a proof-of-concept demonstration of the device design, this work has been done with CVD graphene; however, it is well known that exfoliated graphene exhibits a stronger photoresponse, and the responsivity is expected to exhibit a significant increment if exfoliated graphene is used in combination with the proposed device design. By utilizing a similar approach, a recent work^9^ demonstrated an enhanced graphene photoresponse in the THz range, where a pair of split gates acted simultaneously as a gating control and a plasmonic resonator. In contrast to ref. ^9^, our study demonstrates enhanced performance in another technologically important wavelength range—the visible spectrum. Our study also reveals critical details of the correlation between the optical absorption (thereby heating) and graphene photoresponse.

To compare our work with the earlier reported enhanced graphene photodetectors, we list the key metrics of our work and other relevant works in Table 1 (note: the noise-equivalent power (NEP) for each work is calculated using the formula taken from ref. ^9^). It can be concluded that although the absolute responsivity (which is highly dependent on the quality of graphene and usually peaks for exfoliated graphene) of our device is not the highest, the obtained enhancement factor outperforms the other demonstrations. In Table 2, we compare our work with select works on a wide variety of photodetectors with various materials and mechanisms. We note that our photodetector can be used over an ultrawide range of wavelengths (although only the visible range is reported here). This is because the PTE effect is wavelength independent. In contrast, other mechanisms are often limited to a certain bandwidth. Overall, our device could provide a critical solution for niche applications where good responsivity, a broad operational bandwidth, and a small device footprint are required.

**Table 1:** Comparison of different designs and mechanisms to enhance the graphene photoresponsivity

	Responsivity (mA/W)	NEP (nW Hz^−1/2^)	Frequency range	Enhancement factor	Mechanism
This Work	0.25	11	Visible	25X	Plasmonics + PTE
Castilla et al.^9^	12	0.08	THz		Plasmonics + PTE
Freitag et al.^42^	0.008		Mid-IR	10X	Plasmonics + PTE
Echtermeyer et al.^16^	10	0.64	Visible	20X	Plasmonics + PTE
Liu et al.^18^	6.1	0.64	Visible	5X	Plasmonics + PTE
Brenneis et al.^35^	3 × 10^−4^	6783	Near-IR		PTE
Sun et al.^6^	0.003	88	Near-IR		PTE
Schuler et al.^13^	35		Near-IR		Waveguide + PTE

*NEP* noise-equivalent power, *PTE* photothermoelectric, *IR* infrared

**Table 2:** Performance comparison of photodetectors made with different materials

	Responsivity (mA/W)	Materials	Device thickness	Wavelength range	Mechanism
This Work	0.25	Graphene	150	Visible	PTE
Knight et al.^43^	0.01	Au + ITO	<100 nm	Near-IR	Hot electrons
Michel et al.^44^	1000	Ge + Si	~1 µm	Near-IR	Photovoltaic
Perea-Lopez et al.^45^	0.02	WS_2_	<10 nm	Visible	Excitons
O’Brien et al.^46^	0.4	F8T2 nanowire	15 µm	Visible	Photoconductivity
Thorlabs^47^	440–725	Si	150 µm–10 mm (ACTIVE AREA)	Visible	P-I-N junction
	950–1300	InGaAs	120 um-2 mm	NIR	P-I-N junction
	850–950	Ge	3–10 mm	NIR	P-I-N junction
	120	GaP	2.2 mm	UV	P-I-N junction
Hamamatsu^48^	220–720	Si	1.1–14 mm	Visible	P-I-N junction

*PTE* photothermoelectric, *ITO* indium tin oxide, *IR* infrared, *F8T2* poly[(9,9-dioctylfluorenyl-2,7-diyl)-co-(bi- thiophene)]

As the PTE effect has been shown to be the dominant mechanism of photocarrier generation in graphene, we propose a new approach to enhance the graphene photocurrent by spatially overlapping plasmon-induced optical heating and a p-n junction. We design a novel metal/dielectric/nanodisk trilayer device architecture that includes a gap plasmon structure and a pair of split gates to simultaneously realize enhanced plasmon-induced optical heating and enhanced p-n junction control, respectively. Specifically, the bottom metallic layer (with a gap in the centre) serves as the back reflector of the gap plasmon structure, as well as the electrodes of the split gates. The middle dielectric layer constitutes the optical spacer for the gap plasmon structure and the gating dielectric of the split gates to create a p-n junction. Graphene is then placed on the dielectric layer, followed by deposition of the nanodisk array. Although the nanodisks do not play a role in electrical control, they are an essential part of the gap plasmon structure and efficiently convert light into localized heating. With rigorous experiments, we have proved the dominance of the PTE effect in graphene photocurrent generation and showed that with optical heating and electrical p-n junction enhancement, the photocurrent exhibits a 25-fold increase compared to the un-enhanced case. A source-drain bias of 2.3 V can further enhance the photocurrent by sevenfold. The overall thickness of such a photodetector is 150 nm, which is much smaller than that of state-of-the-art semiconductor photodetectors, which are several microns thick. In addition, the relatively narrowband optical absorptance of the nanodisk gap plasmon structure makes it possible to control the colour sensitivity using nanodisks with different radii, eliminating the need for an additional colour filter layer when integrated into cameras. Last, we note that although photodetectors made with exfoliated graphene usually exhibit higher responsivities^9,13^, they are not suited for large-scale manufacturing owing to the limited size and yield of exfoliated graphene. Our work indicates optimal responsivities that are achievable with CVD graphene, which holds promise for mass production and commercialization. The proposed design represents a leap forward in realizing graphene’s potential in constructing ultrathin, lightweight, and ultrafast photodetectors.

## Materials and methods

### Sample fabrication

The substrate supporting the graphene photodetectors was chosen to be 0.7 mm thick float glass (SiO_2_) because of its low thermal conductivity to create a relatively high temperature profile on the photodetector surface under laser irradiation, which is conducive to photocurrent enhancement (see the main text). Prior to fabrication, the substrate was sonicated in toluene, acetone, and isopropyl alcohol (IPA) for 5 min each. First, alignment marks were created on the substrate using photolithography, gold (Au) metallization and lift-off. Then, the glass substrate was coated with 20 nm thick chromium (Cr) as a charge dissipation layer for the subsequent EBL step, and 200 nm thick poly(methyl methacrylate) (PMMA) was spin-coated on the Cr layer, followed by 5 min of baking on a hot plate set at 180 °C. EBL and 50 s development in methyl isobutyl ketone:IPA 1:3 was performed on the sample to define the geometry of the aluminium (Al) split gates in PMMA, leaving the underlying Cr exposed in areas that would become Al pads in the final device. Then, the sample was rinsed in Cr etchant CR-16 (KMG Electronic Chemicals, Inc.) for 20 s to remove the exposed Cr, followed by a gentle rinse in DI water. In this step, the 150 nm wide and 30 μm long PMMA strip (which would later become the central gap between the split gates) was very susceptible to the CR-16 rinse time and a strong nitrogen stream; thus, after the gentle DI water rinse, the sample was left in air to dry instead of being subjected to more aggressive nitrogen gun blowing. The sample was then transferred to an electron beam evaporator for Ti (5 nm) and Al (100 nm) deposition and soaked in heated (80 °C) acetone overnight for lift-off to remove Ti/Al in the unwanted areas and define the split gates. After this, the sample was rinsed in CR-16 again to completely etch away the Cr that remained on the sample. Ti (5 nm)/Au (100 nm) electrodes were formed in contact with the Al split gates using photolithography, metallization, and lift-off. To ensure electrical contact between Au and Al, the sample was dipped in buffered oxide etch 1:6 for 5 s before the metallization step to remove the native oxide on Al. Then, 20 nm thick Al_2_O_3_ was grown on the Al split gates using ALD as the dielectric spacer/gating dielectric layer (see the main text for a description of this layer). Because Al_2_O_3_ grows preferentially on Al, the Au electrodes were not covered in continuous Al_2_O_3_ after this step, which is favourable for wire bonding during the later electrical test. Graphene was grown by CVD on copper foil^41^, transferred onto the sample using the wet transfer technique, and subsequently defined into smaller (100 μm × 50 μm) rectangles on the split gates using photolithography and O_2_ plasma etching. EBL, Ti (5 nm)/Al (30 nm) metallization, and lift-off were used again to create nanodisk arrays on the graphene sheet. During the lift-off process, some nanodisks were removed from graphene owing to the poor adhesion between Ti/Al and graphene. Finally, photolithography, Ti (5 nm)/Au (100 nm) metallization, and lift-off were used again to make the source and drain contacts to the graphene sheet. The finished sample was wire bonded to a chip carrier and placed in a vacuum chamber for characterization. See Supplementary Fig. S1 for a simplified process flow of the fabrication steps.

### Optical characterization of gap plasmon structures

During the fabrication of the graphene photodetector, we also fabricated a separate sample solely for optical absorptance characterization because the optical characterization technique (WVASE ellipsometer) requires an area of 500 μm × 500 μm of uniform nanodisks to accommodate the incident spot size. The sample consisted of universally 100 nm thick Al and Al_2_O_3_ layers (i.e., these layers were no longer defined in the split gate shape but covered the entire substrate) and nanodisk arrays with disk radius *R* = 50 nm or *R* = 60 nm. We did not include graphene in the optical samples, which resulted in better uniformity of the nanodisks. A variable angle spectroscopic ellipsometer (J.A. Woollam, WVASE) with linearly polarized incident light was used to measure the reflectance (Ref) spectra on the nanodisk arrays. As transmittance is prohibited by the optically thick Al back reflector and scattering is negligible (reflectance is <0.07% for all non-specular angles at all wavelengths, verified independently with an ellipsometer scatterometry measurement), we calculated the absorptance spectra as Abs = 1 − Ref and show them in Fig. 2d of the main text. As a comparison, we also measured an area with only Al and Al_2_O_3_ layers and no nanodisks. Evidently, from Fig. 2d of the main text, the nanodisks significantly enhance the optical absorptance and comprise an essential part in enhancing the PTE current in graphene.

## Supplementary information

Supplementary Information

## Data Availability

The authors declare that all the data supporting the findings of this study are available within the paper and its Supplementary Information.
