# Supplementary material for: Enhancing the graphene photocurrent using surface plasmons and a p-n junction
Source: Light Sci Appl. 2020 Jul 20;9:126. doi: 10.1038/s41377-020-00344-1 (PMC7371713; doi:10.1038/s41377-020-00344-1)
Supplement: Supplementary file 1 — Supplementary Information [file 41377_2020_344_MOESM1_ESM.docx]

Supplementary Information for

“Enhancing the graphene photocurrent
using surface plasmons and a p-n junction”

*Di Wang^1,2^, Andres E. Llacsahuanga Allcca^2,3^, Ting-Fung Chung^2,3^, Alexander V. Kildishev^1,2,4^, Yong P. Chen^1,2,3,4^, Alexandra Boltasseva^1,2,4*^, and Vladimir M. Shalaev^1,2,4*^*

^1^School of Electrical and Computer Engineering, Purdue University, West Lafayette, Indiana 47907, United States

^2^Birck Nanotechnology Center, Purdue University, West Lafayette, Indiana 47907, United States

^3^Department of Physics and Astronomy, Purdue University, West Lafayette, Indiana 47907, United States

^4^Purdue Quantum Science and Engineering Institute (PQSEI), Purdue University, West Lafayette, Indiana 47907, United States

^*^Correspondence and requests for materials should be addressed to A.B. and V.M.S.

# Fabrication process flow of the graphene photodetector

To help visualize the fabrication process of the graphene photodetector described in the Materials and Methods section of the main text, we created the following simplified process flow.


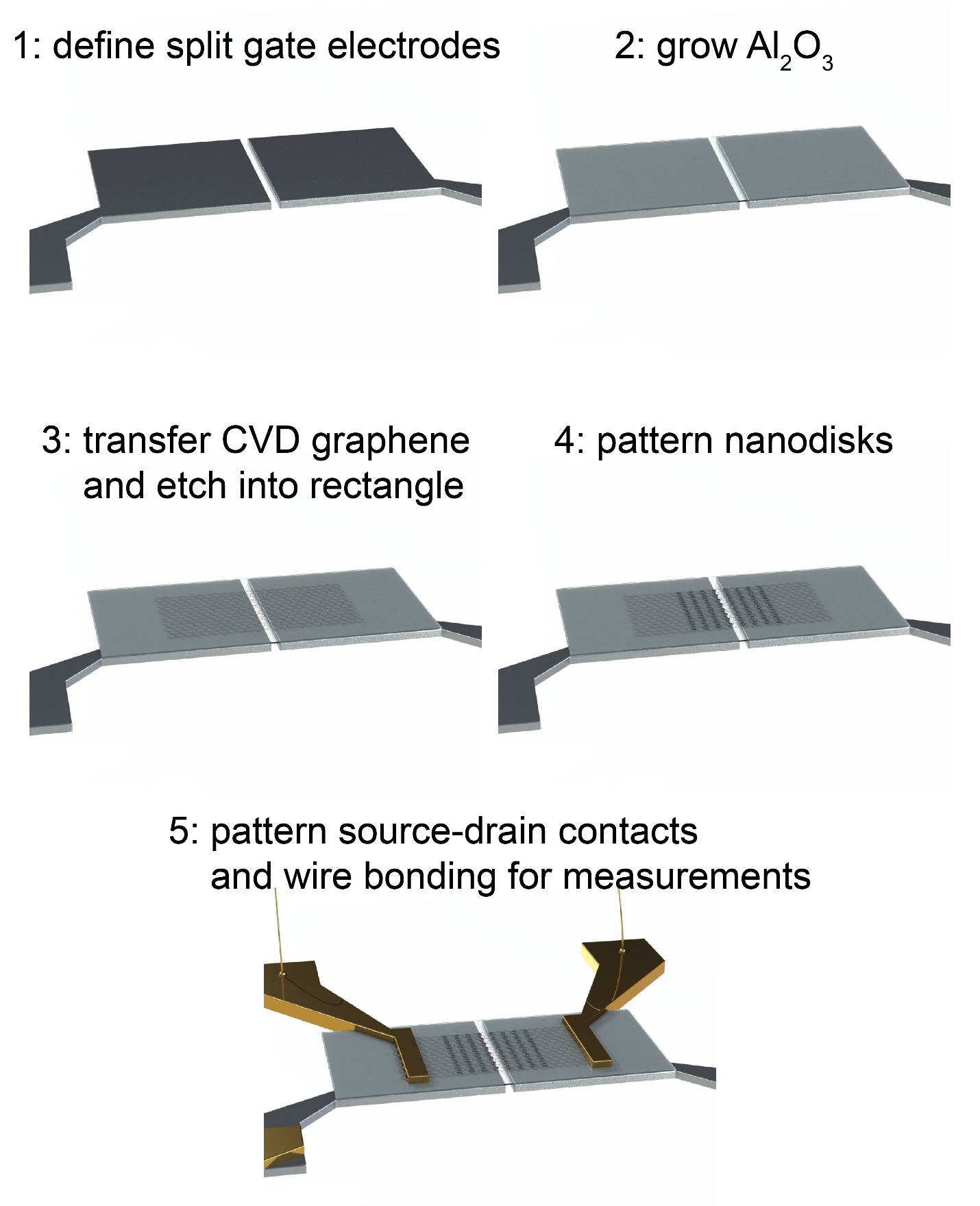


**Figure S1 | A simplified schematic process flow for the fabrication of the graphene photodetector.**

# Responsivity maps versus optical absorptance

As stated in the main text, we obtained the average responsivity versus optical absorptance (Figure 4b of main text) by doing measurements with different combinations of laser wavelength and disk radii. We first measured photocurrent maps like the one shown in Figure 3d, and divided them by the incident optical power to get the responsivity maps shown in Figure S2. The data points shown in Figure 4b of main text are obtained by calculating the mean value of responsivities in the boxed region of each map. The responsivity map of the highest point in Figure 4b of main text is not shown here because its photocurrent map is already shown in Figure 3d of main text.


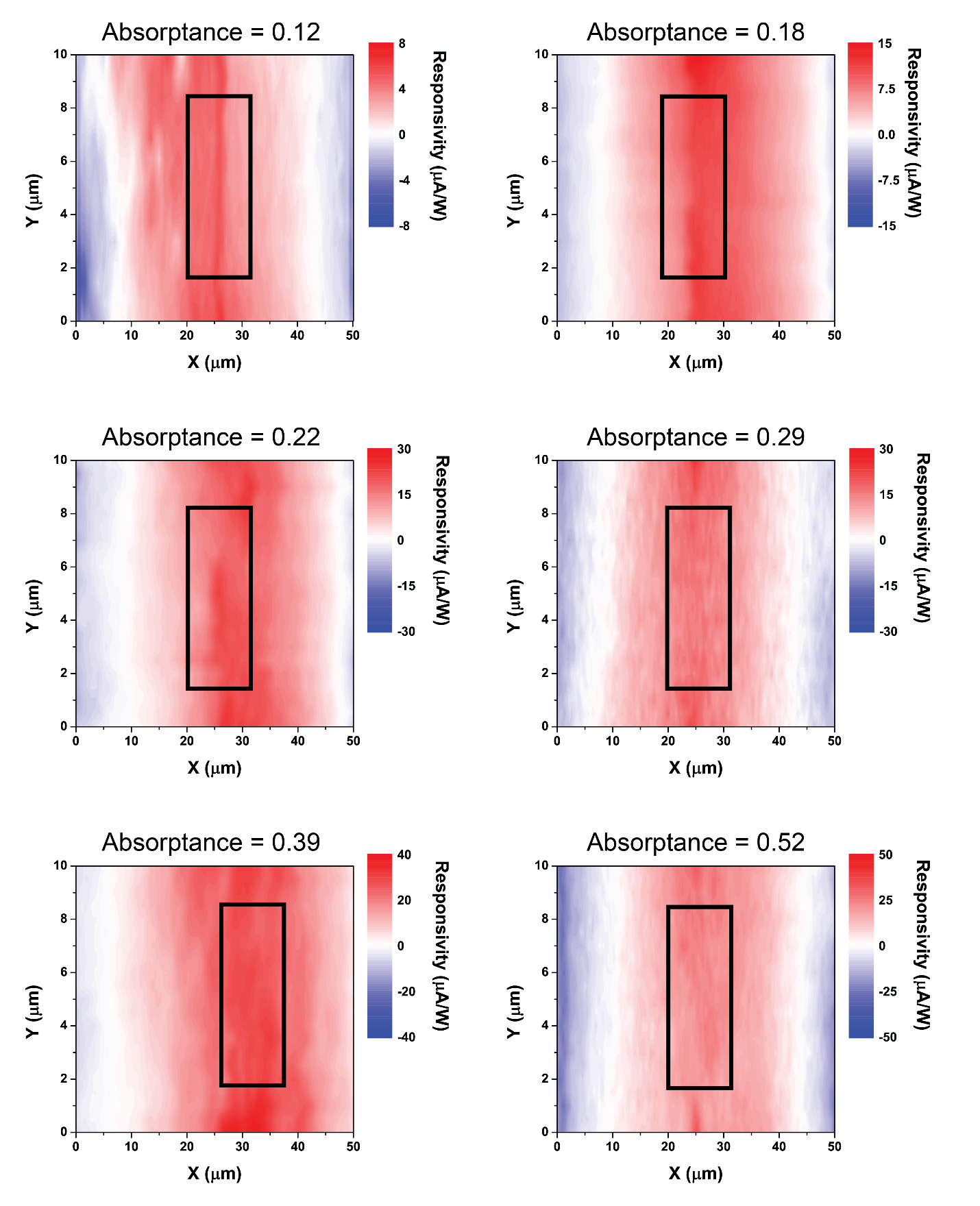


**Figure S2 | Responsivity maps of varying optical absorptance.** The mean values inside the boxed regions are calculated to obtain the individual data points in Figure 4b of the main text.

# Power dependence of photocurrent

We measured the power dependence of photocurrent up to 1.7 mW (32 μW was used in the measurement of Figure 3d of main text). As can be seen from Figure S3, the photocurrent exhibits a linear relationship with the input optical power ($I_{PTE}=10.603\cdot P_{in}$), indicating that the photodetector operates in the weak heating regime where the optically induced change in electron temperature $\left( {\Delta T}_{el} \right)$ is much smaller than the ambient temperature ($T_{o}$). This also shows that the upper bound of the dynamic range is higher than 1.7 mW.


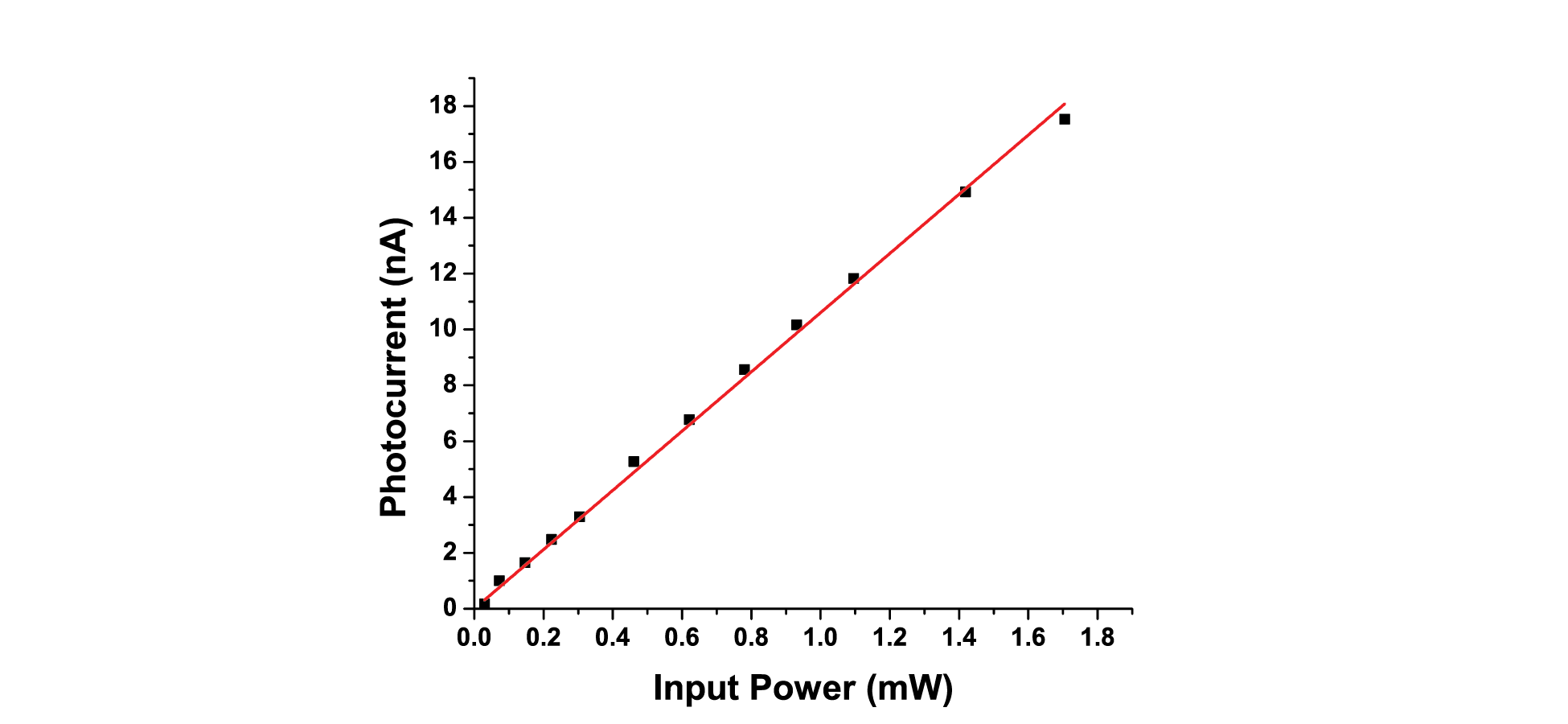


**Figure S3 | Measured photocurrent versus input laser power (black squares) and their linear fit (red line).** The wavelength used in this measurement is 825 nm and the device is loaded with R = 60 nanodisks, resulting in an optical absorptance of 0.29.

# Measurement conditions of Fig. 4b of main text

| Disk Radius (nm) | Wavelength (nm) | Optical Absorptance | Responsivity (µA W^-1^) |
| --- | --- | --- | --- |
| N/A | 532 | 0.12 | 5 |
| 50 | 825 | 0.18 | 11.27 |
| 50 | 638 | 0.22 | 18.79 |
| 60 | 825 | 0.29 | 16.4 |
| 50 | 532 | 0.39 | 25.5 |
| 60 | 532 | 0.52 | 25.21 |
| 60 | 638 | 0.71 | 51.99 |

**Table S1 | Experimental conditions of the data points shown in Fig. 4b of the main text.**

# Numerical modelling of graphene

We use finite element method (FEM, COMSOL Multiphysics, Wave Optics Module) to numerically model the optical response of the graphene-gap-plasmon hybrid system. Graphene is simulated as a volumeless surface current using the method proposed by Emani *et al.*^1^, with a carrier density of 1.42 × 10^13^ cm^-2^, and the surface conductivity of graphene is calculated from Koppens *et al*.^2^ The wavelength in the calculation is set to 638 nm. In the one unit cell simulation, perfect electric (magnetic) conductor boundary condition is used on the outer surfaces that are perpendicular to the electric (magnetic) field, to simulate the case of periodic replication of the unit cell in the x (and y) direction. As a result, the calculated total absorption in the system is 0.91. The optical absorption in bare graphene is estimated using the following formula:$P_{\mathrm{graphene}}=\frac{1}{2}\mathfrak{R}\left( J_{sx}E_{x}^{*}+J_{sy}E_{y}^{*} \right)$, where $J_{sq}$, and $E_{q}$ are the Cartesian components (q∈{x,y}) of the surface current and the tangential electric field inside the graphene sheet respectively.

Since $P_{\mathrm{graphene}}$ is calculated to be 0.13 of the total amount of input power to the system, we determined that the fraction of power dissipated in graphene is 14.4% of the total power dissipation (0.13/0.91). This value is about 7× higher than the optical absorption in a stand-alone graphene sheet (2.3%) without plasmonic enhancement.


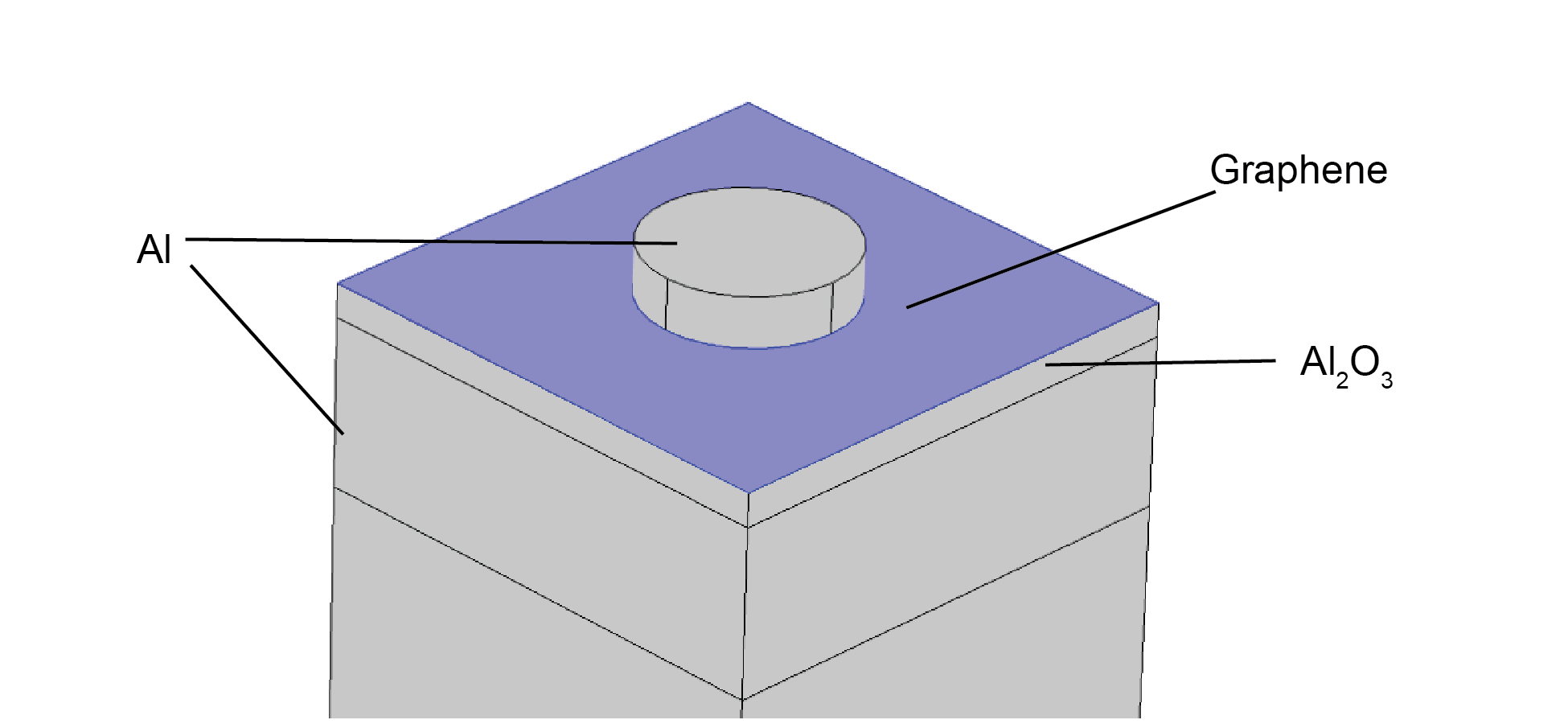


**Figure S4 | Illustration of the numerical model used to simulate the optical absorption in graphene.**

# References

1. Emani, N. K. *et al.* Plasmon resonance in multilayer graphene nanoribbons. *Laser Photon. Rev.* **9**, 650–655 (2015).

2. Koppens, F. H. L., Chang, D. E. & García de Abajo, F. J. Graphene Plasmonics: A Platform for Strong Light–Matter Interactions. *Nano Lett.* **11**, 3370–3377 (2011).
